# Supplementary material for: Effective removal of mercury from aqueous streams via electrochemical alloy formation on platinum
Source: Nat Commun. 2018 Nov 19;9:4876. doi: 10.1038/s41467-018-07300-z (PMC6242894; doi:10.1038/s41467-018-07300-z)
Supplement: Supplementary file 1 — Supplementary Information [file 41467_2018_7300_MOESM1_ESM.pdf]

Effective Removal of Mercury from Aqueous Streams via Electrochemical Alloy Formation on Platinum

Cristian Tunsu* and Björn Wickman**

Chalmers University of Technology, 41296 Göteborg, Sweden

**Department of Chemistry and Chemical Engineering, Nuclear Chemistry and Industrial Materials Recycling*

***Department of Physics, Chemical Physics. Corresponding author, bjorn.wickman@chalmers.se, +46(0)317725179*

Supplementary Discussion

Electrochemical treatment of mercury-containing aqueous solution. Supplementary Figure 1 shows the cyclic voltammograms obtained in 1 mol L^{-1} nitric acid solution, and in 1 mol L^{-1} nitric acid solution containing 10 mg L^{-1} divalent mercury, respectively, in an electrochemical cell comprising a 100 nm platinum nano-film working electrode, a Hg/Hg_2SO_4 reference electrode (REF), and a platinum wire counter electrode. Cycling was done between -0.7 V and 0.98 V vs. REF with a scan rate of 25 mV s^{-1}, under nitrogen bubbling. A working potential of -0.5 V vs. REF was chosen to carry out mercury retrieval studies, which corresponds to a potential of 0.16 vs. the reversible hydrogen electrode (RHE). The current recorded during electrochemical retrieval of mercury from 1 mol L^{-1} nitric acid solution containing 10 mg L^{-1} mercury is presented in Supplementary Figure 2. Values in the range of 40 µA were recorded throughout the experiment.

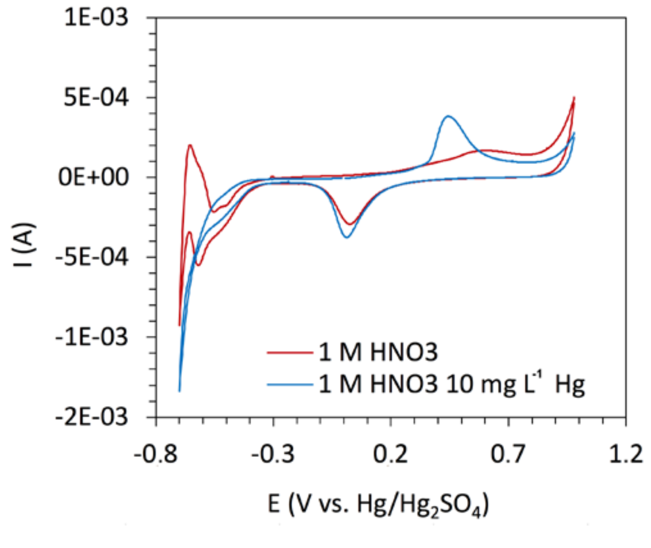

Supplementary Fig. 1 Cyclic voltammogram data for platinum nano-film electrodes. The voltammograms were recorded in 1 mol L^{-1} nitric acid solution (red line), and in 1 mol L^{-1} nitric acid solution with 10 mg L^{-1} divalent mercury (blue line), under nitrogen bubbling. Working electrode: 100 nm platinum film (2.25 cm^2 area). Counter electrode: platinum wire. Reference electrode: Hg/Hg_2SO_4. Scan rate 25 mV s^{-1} between -0.7 and 0.98 V.

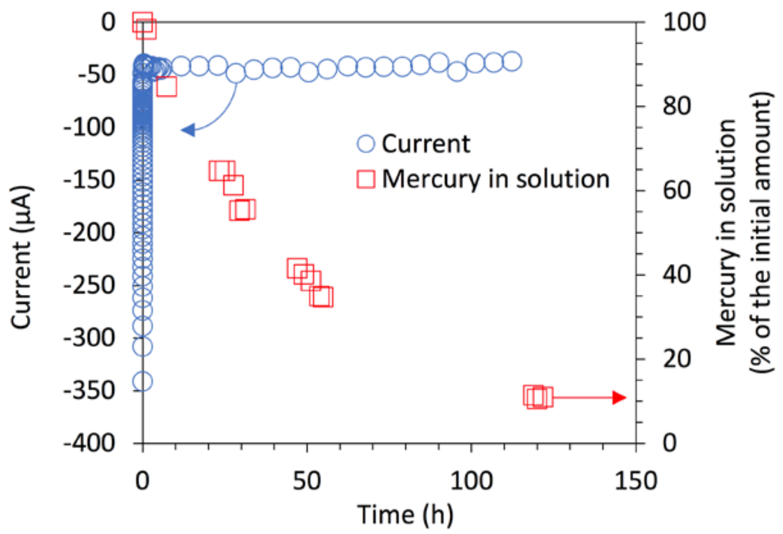

Supplementary Fig. 2 Electrochemical retrieval of mercury from 1 mol L^{-1} nitric acid solution containing 10 mg L^{-1} mercury. The current is plotted on the left axis (blue circles) and the amount of mercury in solution is plotted on the right axis (red squares). Working electrode: 100 nm platinum film (2.25 cm^2 area). Counter: platinum wire. Reference: Hg/Hg_2SO_4. Working potential = 0.16 vs. RHE.

Saturation of a 100 nm platinum nano-film. About 25 % of platinum atoms in the nano-film electrodes (2.25 cm^2, 100 nm platinum thickness) are stoichiometrically needed to completely retrieve mercury as $PtHg_4$ from a solution containing 10 mg L^{-1} mercury. Assuming complete uptake, a solution containing more than 40 mg L^{-1} mercury will saturate the electrode. A retrieval experiment was carried out for 48 h with a solution containing 75 mg L^{-1} mercury, well above the theoretical $PtHg_4$ saturation limit. The results showed that the 100 nm platinum film can be fully saturated to $PtHg_4$. Moreover, upon reaching saturation mercury continues to reduce and deposit on the alloy layer formed (Supplementary Figure 3). For practical applications, this has downsides. Upon saturation, adhesion of the film to the glass substrate decreased, causing the layer formed to crack and peel off. Scanning Electron Microscopy measurements of the cross section of the formed alloy showed a thickness of about 750 nm (Supplementary Figure 3). This is in good agreement with the 760 nm value expected for full transformation of a 100 nm Pt film to $PtHg_4$.

pH dependency. The formation of the $PtHg_4$ alloy from metallic platinum and divalent mercury ions in solution occurs according to Supplementary Equations 1 – 3.

$$\mathrm{Hg^{2+} + 2\,e^- \rightarrow Hg^0} \quad (1)$$

$$\mathrm{Pt + 4\,Hg \rightarrow PtHg_4} \quad (2)$$

$$\mathrm{Pt + 4\,Hg^{2+} + 8\,e^- \rightarrow PtHg_4} \quad (3)$$

As protons and hydroxyl ions are not involved in these equations, the alloying process should not be influenced by the pH of the mercury-containing solution. At different pH values, the platinum surface will be different at a certain potential relative to the standard hydrogen electrode (SHE). This is due to the fact that protons are adsorbed on the platinum surface at low potentials, and hydroxyl ions are adsorbed on platinum at higher potentials[1]. These processes are directly dependent on pH. In fact, the

platinum surface can be either metallic, proton-covered or hydroxyl-covered at a certain potential vs. the SHE, depending on the pH of the solution. To avoid influences from changes on the platinum surface, the pH dependence of mercury alloy formation was done at a fixed potential vs. the reversible hydrogen electrode (RHE). According to the aforementioned cyclic voltammetry data, alloy formation from 1 mol L^{-1} nitric acid solution (pH 0) occurs at -0.5 V vs. REF. This corresponds to 0.16 V vs. SHE or RHE. For subsequent retrieval experiments at other pH values, the working potential was 0.16 V vs. RHE. As derived from the Nernst equation, the dependency of the RHE scale on pH is given by Supplementary Equation 4.

$$E_{RHE} = E_{SHE} + 0.059\ pH \qquad (4)$$

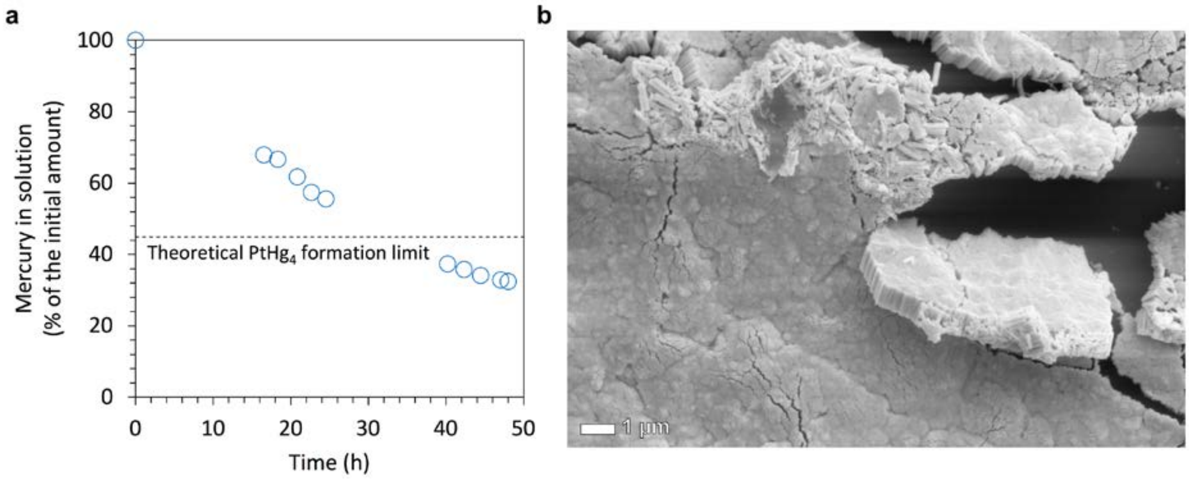

Supplementary Fig. 3 Saturation of a 100 nm platinum electrode. **a** Decrease of mercury concentration in 50 mL solution 1 mol L^{-1} HNO3 containing 75 mg L^{-1} mercury. Working electrode: 100 nm platinum film (2.25 cm^2 area). Counter: platinum wire. Reference: Hg/Hg_2SO_4. Working potential = 0.16 vs. RHE. **b** Scanning Electron Microscopy picture of the formed alloy. Upon saturation, adhesion of the metallic film to the glass substrate decreased, causing the layer formed to crack and peel off. Measurements of the cross section of the alloy showed a thickness of about 750 nm.

The influence of temperature on the mercury retrieval process. It was mentioned in the manuscript that, after the first layers of alloy is formed, additional mercury needs to penetrate into the metallic alloy film to grow the alloy. This diffusion of mercury is a slower process and likely the rate determining step during retrieval. To verify this hypothesis, we have performed retrieval studies at ambient conditions, 20 ± 1 °C, and at higher temperature, 40 ± 1 °C. As diffusion becomes faster at higher temperature, the results will show if the retrieval process is limited by the formation of thicker alloy layers. Supplementary Figure 4 shows the results for a test performed on electrolytes containing 10 mg L^{-1} divalent mercury dissolved in 1 mol L^{-1} nitric acid. As seen, retrieval was significantly faster, which confirmed our hypothesis.

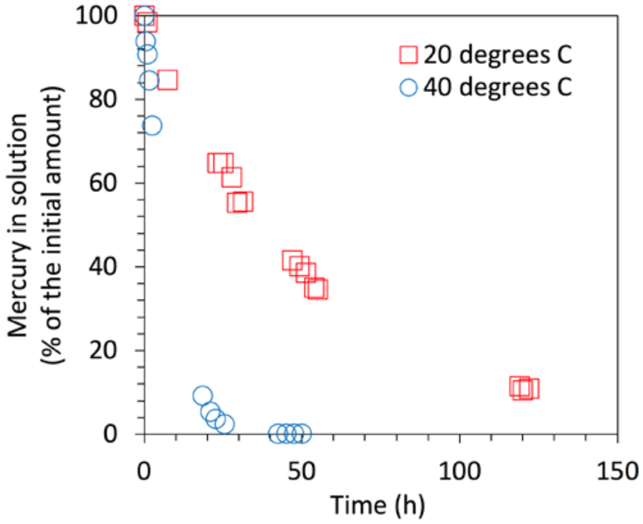

Supplementary Fig. 4 The influence of temperature on alloy formation. Electrochemical retrieval of mercury was done from 1 mol L^{-1} nitric acid solution containing 10 mg L^{-1} mercury at two different temperatures, 20 ± 1 °C (red squares) and 40 ± 1 °C (blue circles). Working electrode: 100 nm platinum film (2.25 cm^2 area). Counter: platinum wire. Reference: Hg/Hg_2SO_4. Working potential = 0.16 vs. RHE.

Supplementary Note

As mentioned in the main article, common methods to remove mercury ions from solution include precipitation, solvent extraction and ion exchange. Precipitation has limitations when it comes to large volumes of solution containing very low amounts of mercury, and requires further effective separation of the formed precipitate to isolate the contamination from the stream. Solvent extraction and ions exchange techniques overcome these drawbacks but may also pose limitations. During solvent extraction, undesired dissolution and dispersion of organic compounds in the aqueous feed can occur[2]. For such reasons, the use of organic extractants dangerous to aquatic life may not be suitable to decontamination water streams hosting aquatic life or streams that are later discarded to natural waters. Ion exchange and also solvent extraction are not suited for waters which contain solids or dispersed particles. Regeneration and/or stripping of extractants and ion exchange resins requires additional chemical steps, which can generate secondary wastes. The use of adsorbents containing thiol groups (-SH) as active sites for binding mercury is not suitable for contaminated oxidizing solutions. Under such conditions, thiol groups are oxidized to disulfide, affecting the binding of mercury, and the chemical structure of the absorbent[3]. pH also plays an important role in the decontamination via precipitation, ion exchange and solvent extraction. Depending on the system, the pH of the stream may need adjustment to facilitate effective decontamination, not always feasible for very acidic/alkaline solutions. The process presented here does not suffer from such drawbacks.

Supplementary References

1 Pourbaix, M. J. N., Van Muylder, J. & de Zoubov, N. Electrochemical Properties of the Platinum Metals. *Platinum Metals Review* **3**, 47-53 (1959).

2 Ritcey, M. G. in *Solvent extraction principles and practice, second edition, revised and expanded* (eds J. Rydberg, M. Cox, C. Musikas, & G.R. Choppin) 277-338 (Marcel Dekker, 2004).

3 Ebadian, M. A. *Mercury contaminated material decontamination methods: investigations and assessment*. 42 (National Energy Technology Laboratory, 2001). <http://www.osti.gov/bridge/servlets/purl/790964-1xmGv6/native/>
